# “HIIT the Inflammation”: Comparative Effects of Low-Volume Interval Training and Resistance Exercises on Inflammatory Indices in Obese Metabolic Syndrome Patients Undergoing Caloric Restriction

**DOI:** 10.3390/nu14101996

**Published:** 2022-05-10

**Authors:** Dejan Reljic, Walburga Dieterich, Hans J. Herrmann, Markus F. Neurath, Yurdagül Zopf

**Affiliations:** 1 Hector-Center for Nutrition, Exercise and Sports, Department of Medicine 1, University Hospital Erlangen, Friedrich-Alexander University Erlangen-Nürnberg, 91054 Erlangen, Germany; walburga.dieterich@uk-erlangen.de (W.D.); hans.herrmann@uk-erlangen.de (H.J.H.); yurdaguel.zopf@uk-erlangen.de (Y.Z.); 2 German Center Immunotherapy (DZI), University Hospital Erlangen, Friedrich-Alexander University Erlangen-Nürnberg, 91054 Erlangen, Germany; markus.neurath@uk-erlangen.de; 3 Department of Medicine 1, University Hospital Erlangen, Friedrich-Alexander University Erlangen-Nürnberg, 91054 Erlangen, Germany

**Keywords:** obesity, cardiometabolic health, CRP, high-intensity interval training, single-set resistance training, whole-body electromyostimulation

## Abstract

Exercise is a cornerstone in metabolic syndrome (MetS) treatment. However, the effects of low-volume exercise modalities on MetS-associated low-grade inflammation are unclear. A total of 106 MetS patients (53.7 ± 11.4 years) were randomized to low-volume high-intensity interval training (LOW-HIIT, 14 min/session), single-set resistance training (1-RT, ~15 min/session), whole-body electromyostimulation (WB-EMS, 20 min/session), three-set resistance training (3-RT, ~50 min/session), each performed 2 ×/week for 12 weeks, or a control group (CON). All groups received nutritional counseling for weight loss. Inflammatory and cardiometabolic indices were analyzed pre- and post-intervention. All groups significantly reduced body weight by an average of 3.6%. Only LOW-HIIT reduced C-reactive protein (CRP) (−1.6 mg/L, *p* = 0.001) and interleukin-6 (−1.1 pg/mL, *p* = 0.020). High-sensitivity CRP and lipopolysaccharide-binding protein decreased following LOW-HIIT (−1.4 mg/L, *p* = 0.001 and −2.1 ng/mL, *p* = 0.004) and 3-RT (−0.6 mg/L, *p* = 0.044 and −2.0 ng/mL, *p* < 0.001). MetS severity score improved with LOW-HIIT (−1.8 units, *p* < 0.001), 1-RT (−1.6 units, *p* = 0.005), and 3-RT (−2.3 units, *p* < 0.001). Despite similar effects on body weight, low-volume exercise modalities have different impact on inflammatory and cardiometabolic outcomes in MetS patients. LOW-HIIT has superior efficacy for improving inflammation compared to 1-RT and WB-EMS. Resistance-based exercise appears to require a higher volume to promote beneficial impact on inflammation.

## 1. Introduction

During the last decades, the prevalence rates of obesity have continued to rise in most countries all over the world [1]. Excess body weight with an excessive accumulation of adipose tissue is associated with an increased risk of developing numerous chronic diseases [2]. If obesity is accompanied by other cardiometabolic risk factors, such as hypertension, high storage of visceral fat, hyperglycemia, or dyslipidemia (i.e., the metabolic syndrome, MetS), the risk of serious health issues and premature mortality is further substantially increased [3,4]. During the ongoing COVID-19 pandemic, it has also been observed, for example, that patients with preexisting obesity and cardiometabolic risk factors exhibit an increased likelihood for a severe or fatal disease course after a SARS CoV-2 infection [5,6,7], which currently illustrates the aggravated health risk in obese MetS populations.

Obesity and MetS are typically associated with chronic low-grade inflammation, which in turn can contribute to the development of multiple secondary diseases, including cardiovascular disease (CVD) and several cancer types [8,9]. The underlying mechanisms by which obesity and MetS promote chronic inflammation are not yet fully understood but it is suggested that a dysregulated and excessive secretion of proinflammatory adipokines and cytokines from adipose tissue play a critical role in pathogenesis [8,9,10,11,12]. Given the deleterious health consequences of chronic inflammatory conditions, reducing inflammation is an important target in obesity and MetS treatment. Dietary modifications, in particular caloric restriction [13,14] and regular physical exercise [15,16], are essential elements in the treatment of obesity and associated cardiometabolic disorders. Previous research in both rodents and humans indicates, for example, that caloric restriction may be a potent strategy to downregulate inflammation [17]. While inflammatory markers may increase following an acute exercise bout [18], it has been shown that longer-term aerobic [19,20] and resistance [20,21] training can have a beneficial impact on the reduction of chronic inflammation.

However, despite the well-established health benefits of exercise, a large proportion of adults across the globe [22], in particular obese individuals [23], do not achieve the recommended amounts of weekly physical activity (i.e., at least 150 min of moderate or 75 min of vigorous aerobic physical activity per week plus 2–3 weekly sessions of muscle-strengthening exercises [24]), with insufficient time being cited as a major obstacle to regular exercise [25,26]. In this context, various time-efficient exercise modalities, such as low-volume high-intensity interval training (HIIT) [27,28,29], low-volume (single-set) resistance training (RT) [30], or whole-body electromyostimulation (WB-EMS) [31] have gained growing popularity in recent years. The evaluation of the health benefits that can be achieved with these low-volume exercise modalities is an ongoing focus of research. HIIT is a specific type of cardiovascular training consisting of alternating exercise and recovery periods of various durations [27]. “Low-volume” HIIT (LOW-HIIT) is a very time-efficient subtype of interval training that involves per previous definition a cumulative duration of ≤10 min of vigorous exercise intervals within a session of ≤30 min total duration (including warm-up, recovery periods, and cool-down) [28]. There is increasing evidence that LOW-HIIT protocols can evoke comparable or even greater improvements in several health-related outcomes, including maximal oxygen uptake (VO_2max_) and markers of glycemic control [32,33] as traditional moderate-intensity continuous training (MICT), despite significantly lower time effort. Recently, we [34,35] and others [36] have also shown that LOW-HIIT is feasible and effective to improve cardiometabolic risk indices in obese patients with MetS.

In addition to exercise modalities such as HIIT and MICT that primarily target to improve cardiorespiratory fitness [15,37], regular RT is advocated to maintain and improve skeletal muscle mass and muscle strength [15,38]. Most RT programs typically involve multiple sets of each exercise in a workout. However, research reports, including a recent study from our group [39], have documented that single-set RT (1-RT) protocols may (at least initially) evoke similar health benefits as multiple-set RT programs in previously sedentary populations and various clinical cohorts [30,40,41]. Compared to multiple-set RT workouts (mostly consisting of 2–3 sets per exercise), 1-RT programs consist of just one set of repetitions per exercise, making them more time-efficient [30].

Furthermore, WB-EMS has emerged in recent years as a very time-efficient resistance-type exercise technology, which has gained growing popularity over the past few years in commercial fitness facilities as it allows a synchronous training of all major muscle groups via electric stimulation using specific exercise suits [42]. While there is increasing evidence for the beneficial effects of LOW-HIIT, 1-RT, and WB-EMS on physical fitness parameters and various cardiometabolic health outcomes, comprehensive data, however, on the impact of very low-volume exercise modalities on chronic inflammation in clinical populations are still lacking.

The purpose of the present investigation was, therefore, to compare the effects of 12 weeks of LOW-HIIT, 1-RT, and WB-EMS on inflammatory and cardiometabolic indices in a cohort of obese patients with MetS undergoing caloric restriction against each other and against two comparative groups, either performing a “traditional” three-set resistance training (3-RT, active, high-volume control group) or an inactive control group (CON). Based on previous research from others [17] and our group demonstrating the beneficial impact of LOW-HIIT [34,35], 1-RT [39], and WB-EMS [43] on body composition (i.e., body fat reduction) and cardiometabolic outcomes, we hypothesized that all low-volume exercise programs would have positive effects on inflammation status, and that caloric restriction alone would be less potent to reduce inflammation than when combined with exercise. Given research findings indicating that cardiovascular-based exercises appear to be more effective for improving chronic inflammation than RT [44], we expected that the anti-inflammatory effects would be larger in the LOW-HIIT group in comparison to the three RT groups. Moreover, given the reported exercise dose-response effects on the reduction of inflammatory markers [44], we assumed that 3-RT would have superior effects on reducing chronic inflammation than the two low-volume RT groups (1-RT and WB-EMS).

## 2. Materials and Methods

### 2.1. Study Design

This study was a sub-analysis combing patient data from two previously published larger randomized-controlled clinical trials [35,39]. In the main trials, patients were randomly assigned to different exercise regimens (including LOW-HIIT, 1-RT, WB-EMS, and 3-RT) performed for a duration of 12 weeks combined with nutritional counseling to support weight loss through caloric restriction, or to inactive control groups only receiving nutritional counseling (standard care). Sample size calculation and randomization procedures of the main trials were previously reported [35,39]. This sub-analysis focused on the impact of the different low-volume exercise programs on inflammation outcomes in a sub-sample of patients. The primary outcome of this study was serum concentration of C-reactive protein (CRP), and secondary outcomes were further inflammatory markers, cardiometabolic indices, VO_2max_, and anthropometric variables as specified below. Based on a recent meta-analysis on the influence of exercise on low-grade systemic inflammation in overweight and obese individuals [44], indicating an average reduction by ~0.84 mg/L of CRP (corresponding to an effect size of d = ~0.43), a sample size calculation suggested that *n* = 90 patients (18 patients per group) would be required for this study to achieve a 90% power in a 2-way repeated measures analysis of variance with five groups and two times of measurement at a significance level of 5% (G*Power, version 3.1.9.2, Heinrich Heine University Düsseldorf, Düsseldorf, Germany). To account for dropout, we prospectively selected a total of *n* = 125 patients from the two main trials for this sub-analysis by random. Patients selected for this sub-analysis received two additional pre- and post-intervention blood samples for the determination of inflammatory markers. As in the main trials, we additionally determined the cardiometabolic risk status quantified using the metabolic syndrome severity score (MetS z-score) for all sub-groups. All patients were fully informed about the objectives and procedures of the study, which was in accordance with the Helsinki Declaration, and provided written consent before enrolment. The study protocols of the main trials were approved by the Ethics Committee of the Medical Faculty of the Friedrich-Alexander University Erlangen-Nürnberg (approval number: 203_17B and 210_17B, respectively) and registered at ClinicalTrials.gov (ID-number: NCT03306056, and NCT03306069).

### 2.2. Patients

Advertisements posted in local newspapers and flyers distributed in surrounding medical practices were used to recruit patients for the study. Eligibility criteria for participation in the main trials were previously described in detail [35,39]. In brief, inclusion criteria were age ≥ 18 years, obesity (body mass index, BMI ≥ 30 kg/m^2^), diagnosis of MetS according to pre-defined criteria [45], and a self-reported predominantly sedentary lifestyle as defined by the American College of Sports Medicine [46]. Exclusion criteria were clinical diagnosis of heart disease, cancer, severe orthopaedic conditions, injuries and illnesses associated with acute inflammation, such as bacterial or virus infections, other conditions that might prohibit safe participation in an exercise program, and pregnancy. All patients agreed to maintain their usual lifestyle during the intervention period to minimize potential bias. Any necessary changes in medications or dosages during the intervention period were only made after medical clearance and in consultation with the investigators.

### 2.3. Health Examinations

The baseline health examination was conducted 1 week before the onset of the intervention. The examination included all study outcome assessments, as further specified below, as well as resting electrocardiography and cardiopulmonary exercise testing (CPET) to assure safe participation in the training programs and to verify that no exclusion criteria (as specified above) are present. The concluding examination was carried out during the first week after completion of the intervention, with a time interval of at least 3 days to the final training session and at an equal day time to assure proper recovery and to minimize potential circadian effects. All procedures and measurements were performed in a controlled laboratory setting with stable ambient conditions at our Research Lab. Patients were asked to arrive overnight-fasted and to sustain from alcohol and vigorous physical activities for at least 24 h before the respective examinations. The outcome assessments were carried out single-blinded, so that the investigators involved in data collection were unaware of the patients’ group assignment.

#### 2.3.1. Measurements of Blood Pressure

First, patients were asked to empty their bladder and to rest seated for a duration of 5 min. Thereupon, measurements of blood pressure were performed using an automatic upper-arm blood pressure device (M5 professional, Omron, Mannheim, Germany), previously proven to provide accurate readings [47]. Two measurements in a row were performed on each arm in intervals of 60 s. Subsequently, the values of the arm with the higher blood pressure were averaged and recorded for further analysis as previously recommended by current guidelines [48].

#### 2.3.2. Blood Collection

Blood samples were collected through venipuncture of the patients’ antecubital arm vein with the help of a disposable cannula (S-Monovette, Sarstedt, Nürmbrecht, Germany). Blood analyses included serum concentrations of the inflammatory markers C- CRP and high-sensitivity CRP (hsCRP) and the cardiometabolic markers fasting glucose, triglycerides, total cholesterol, low-density lipoprotein cholesterol (LDL-C), and high-density lipoprotein cholesterol (HDL-C). Subsequent analyses were conducted at the laboratories of the University Hospital Erlangen as previously described in detail [35,39]. Additionally, the inflammatory markers interleukine-1 beta (IL-1β), interleukine-6 (IL-6), interfereone-gamma (IFNγ), and lipopolysaccharide-binding protein (LBP) were determined at the laboratory of the Hector-Center for Nutrition, Exercise and Sports. IL-1ß, Il-6, IFNγ, and LBP measurements were done with commercially available sandwich enzyme-linked immunosorbent assays (Human IFN-gamma DuoSet ELISA; Human IL-1 beta/IL-1F2 DuoSet ELISA; Human IL-6 Quantikine HS ELISA Kit; Human LBP DuoSet ELISA; all R&D systems) according to manufacturer’s instructions. For all determinations, 100 µL undiluted serum was used and determination was performed in duplicates. Briefly, wells were coated with the corresponding capture antibody over night at room temperature. Three wash steps were followed by blocking with reagent diluent for 1 h at room temperature. Afterwards, 100 µL of undiluted serum samples were transferred in duplicates to the coated wells for 2 h at room temperature. Wells were washed and further incubated with biotinylated detection antibody for 2 h at room temperature. Bound antibodies were detected with Streptavidin-HRP solution for 20 min at room temperature and colour development was done with substrate solution for another 20 min. The assay was stopped with sulfuric acid and the optical density was measured at 450 nm with BioRad iMark^TM^ Microplate Reader. The serum concentrations were determined by point-to-point calculation.

#### 2.3.3. Anthropometric and Body Composition Measurements

For body composition measurements, patients were still in a fasted state and again asked to empty their bladder if necessary. Measurements were performed with a multi-frequency segmental bioelectrical impedance analysis (BIA) device (seca mBCA 515, Seca, Hamburg, Germany), which has been previously shown to yield valid body composition estimates when compared to magnetic resonance imaging (MRI) [49]. Patients’ waist circumference (WC) was obtained in upright position, to the nearest millimeter, using a measuring tape.

#### 2.3.4. MetS z-Score Determination

The MetS z-score represents a risk score that was developed to assess severity of MetS. It has been suggested that MetS z-score is more accurate to express the patient’s overall cardiometabolic risk status compared to individual categorical risk factors (e.g., blood pressure or fasting glucose), which may fail to identify changes of clinical relevance if specific threshold values to move out of a certain (“pathologic”) range are not reached [50]. Calculation of the MetS z-score was performed using the following equations, based on the variables sex (F: females, M: males), waist circumference (WC), mean arterial blood pressure (MAB), fasting serum glucose (GLU), triglycerides (TG), and high-density lipoprotein cholesterol (HDL-C) [51]:

M: [(40 − HDL-C)/9.0] + [(TG − 150)/81.0] + [(FBG − 100)/11.3] + [(WC − 102)/7.7] + [(MAB − 100)/9.1]

F: [(50 − HDL-C)/14.1] + [(TG − 150)/81.0] + [(GLU − 100)/11.3] + [(WC − 88)/9.0] + [(MAB − 100)/9.1]

#### 2.3.5. Cardiopulmonary Exercise Test (CPET)

Patients performed a standardized CPET on an electronically braked cycle ergometer (Corival cpet, Lode, Groningen, The Netherlands). After a brief familiarization period of 1 min, the initial load was set at 50 W and subsequently gradually increased by 12.5 W·per min (1 W every 5 s) for females and 15 W per min (1 W every 4 s) for males, respectively, until volitional exhaustion. Exhaustion was accepted if at least two of the following criteria were reached: oxygen uptake leveling-off, peak respiratory exchange ratio (RER_peak_) ≥ 1.1, age predicted HR_peak_ ≥ 90% (according to the formula: 220–age), and a maximum degree of perceived exertion of ≥ 19 on the Borg scale [52]. HR was recorded continuously in real time with a 12-lead ECG system (custo cardio 110, custo med, Ottobrunn, Germany). Oxygen uptake (VO_2_) and carbon dioxide output (VCO_2_) were determined using an open-circuit breath-by-breath spiroergometric system (Metalyzer 3B-R3, Cortex Biophysik, Leipzig, Germany). All measurements were averaged over every 10 s. CPET data were subsequently used to calculate exercise target HR values for the patients allocated to LOW-HIIT.

#### 2.3.6. Maximum Strength (Fmax) Testing

Patients allocated to RT or WB-EMS additionally performed a modified Fmax test of the main muscle groups (in detail, chest, upper back, abdominals, lower back, and legs) at the baseline examination, after 4 and 8 weeks of training, and post-intervention. While a “real” Fmax test typically aims to detect the one-repetition maximum (i.e., the maximum weight an individual can move for a single repetition), the modified Fmax test used in this study was based on the performance of multiple repetitions. This approach is associated with lower injury risk and thus recommended for untrained cohorts [53]. Initially, patients performed a brief warm-up and familiarized with the test procedures. Subsequently, Fmax tests were conducted under supervision of certified physiotherapists and sports therapists on the following five devices: Chest press, lat pulldown machine, lower back machine, abdominal crunch, and leg press (TechnoGym, Neu-Isenburg, Germany). This standardized order was maintained at each measurement time point. At each device, patients were asked to lift the applied weight load as often as possible until muscle failure. It has been suggested that the repetition number should not exceed six in order to be able to make accurate one-repetition maximum predictions [54]. Thus, in case that more than six repetitions were managed, the weight load was raised and a new a test was executed after a recovery period of 3 min. Typically, the load that could be lifted for a maximum of six repetitions was detected within a maximum of three attempts. Subsequently, Fmax was computed according to the following equation [55]:

Fmax = 100 × load rep/(102.78 × 2.78 × rep)

### 2.4. Nutritional Counseling

All patients obtained comprehensive nutritional counseling from a qualified dietitian in a one-on-one conversation at study entry. Based on international guidelines for obesity treatment [56], patients were advised to achieve a daily caloric deficit of 500 kcal. Additionally, patients were instructed to maintain a proper protein consumption by ≥1.0 g/kg/day in order to prevent a loss of muscle mass during caloric restriction [57]. Furthermore, patients received handouts with recipes and food lists to support adherence to the dietary recommendations. Nutritional intake was analyzed using 3-day food records (Freiburger Ernährungsprotokoll; Nutri-Science, Freiburg, Germany) at study onset and during the final week of the intervention period. Average caloric and macronutrient intakes were evaluated using the software PRODI 6 expert (Nutri-Science, Freiburg, Germany).

### 2.5. Exercise Training Programs

All training sessions were supervised by certified physiotherapists and sports therapists, who were skilled in the implementation of the different exercise protocols. The average therapist-patient ratio was 1:2 during LOW-HIIT and the RT-programs, and 1:1 during WB-EMS, respectively. All exercise groups trained two times per week with a minimum of 2 days of recovery between sessions for a total duration of 12 weeks (24 sessions in total). Patients were free to select their preferred training times individually during the Trainings Center’s opening hours.

#### 2.5.1. Very Low-Volume High-Intensity Interval Training

The low-volume HIIT protocol was in line with the protocol established by Reljic et al. and as previously described in detail [58]. In brief, the LOW-HIIT sessions were performed on electronically braked cycle ergometers (Corival cpet, Lode, Groningen, The Netherlands) and consisted of a 2 min warm-up phase, followed by 5 intervals of 1 min at an exercise intensity corresponding to 80–95% HR_peak_ divided by 1 min recovery periods of low intensity and a concluding 3 min cooldown (14 min total time per session). The minimum exercise intensity (HR) to be reached during the single intervals was progressively elevated according to the following pattern: week 1–4: 80–85% HR_peak_, week 5–8: 85–90% HR_peak_, and week 9–12: 90–95% HR_peak_. Patients were instructed to adjust the pedal cadence and/or the load resistance during each interval bout to reach their individually determined target HR. Patients were equipped with chest strap HR monitors (acentas, Hörgertshausen, Germany), which allowed them to continuously track their HR responses during the training session. HR values of each session were recorded and subsequently evaluated using a specific HR analysis software (HR Monitoring Team System, acentas, Hörgertshausen, Germany).

#### 2.5.2. Resistance Training

Each RT session started with a 5 min warm-up of low intensity ergometer cycling. Subsequently, five exercises were performed to address all main muscle groups (i.e., chest, upper back, abdominals, lower back, and legs) using the following weight-machines: Chest press, lat pulldown machine, lower back machine, abdominal crunch, and leg press (TechnoGym, Neu-Isenburg, Germany). Based on the Fmax values determined baseline and thereafter every 4 weeks, the weight load was progressively elevated over the 12 weeks as follows: 50–60% Fmax during week 1–4; 60–75% Fmax during week 5–8; and 70–80% Fmax during week 9–12. All exercises were performed according to the following pattern: 2 s of concentric (weightlifting phase) and 2 s of eccentric (weight lowering phase) muscle work until fatigue. The 1-RT group performed a single set of each exercise. Total time per exercise session was ~15 min (including the 2 min resting phases between exercises). The 3-RT group performed three sets of each exercise with 2 min rest in between, adding up to a total session time of ~50 min.

#### 2.5.3. Whole-Body Electromyostimulation

Similar to the RT-programs, every training session commenced with a 5 min warm-up on a stationary ergometer. WB-EMS was executed using devices and specific exercise suits (miha bodytec, Gersthofen, Germany), including upper-arm and thigh cuffs, a hip belt, and vest with embedded electrodes to evoke electrical stimulation of the muscles. The electrical stimulation was applied by bipolar impulses at an 85 Hz frequency and a 350 μs pulse width. The electrical stimulation was induced intermittently with a 6 s impulse phase followed by 4 s rest as most frequently practiced in commercial fitness facilities [42]. In total, eight muscle groups were stimulated by the WB-EMS application, including chest, upper back, latissimus, upper arms, abdomen, lower back, buttocks, and thighs. The current intensity was adjusted to evoke a perceptible muscle contraction. During each impulse phase, patients performed two sets of light exercises, including butterfly and pull-down movements with the arms, trunk flexion and extension, and half squats, each repeated for ten times, resulting in a total session time of 20 min. Current intensity was individually adjusted in every single session and, if required, increased to assure a progressive training stimulus.

### 2.6. Statistical Analysis

All analyses were conducted with SPSS version 24.0 (SPSS Inc., Chicago, IL, USA). Initially, data distribution was analyzed using the Shapiro-Wilk test. In case of normal data distribution, a 5 × 2 repeated-measures analysis of variance (ANOVA) was performed to investigate main effects of group and time as well as interaction effects between both factors. Subgroup analyses were performed to check whether gender had any influence on changes in CRP serum concentrations and the MetS z-score. Variance homogeneity was checked with the Levene’s test. If significant main or interaction effects were found, one-way ANOVAs followed by Holm-Sidak’s post hoc tests and post hoc paired *t*-tests were conducted to analyze between-group differences and within-group (pre-post) changes, respectively. If data were not normally distributed, log or square root transformation was performed and the same aforementioned statistical tests were used with the transformed values. If the transformation did not lead to data normalization, the non-parametric Friedman two-way analysis of variance by ranks was performed, and, in case of significant results, followed by Dunn’s Bonferroni post hoc tests for between-group comparisons and Wilcoxon’s and Mann-Whitney tests for within-group post-hoc comparisons. Effect sizes, including partial eta-squared (*ηp*^2^) for ANOVA and Kendall’s coefficient of concordance (*W*) for the Friedman test, were calculated and classified as follows: ≤0.01: small, ≥0.06: medium, and ≥0.14: large for *ηp*^2^, and ≤0.10: small, ≥0.30: medium, and ≥0.50: large for *W*, respectively [59]. Additionally, Pearson (*r*) or Spearman (ρ) correlation analyses were performed to quantify the association between the changes in different outcomes. All data are reported as means ± standard deviation (SD). Pre- and post-intervention changes are displayed with 95% confidence intervals (95% CI). The significance level was *p* < 0.05 for all analyses.

## 3. Results

### 3.1. Study Flow

**Figure 1:**
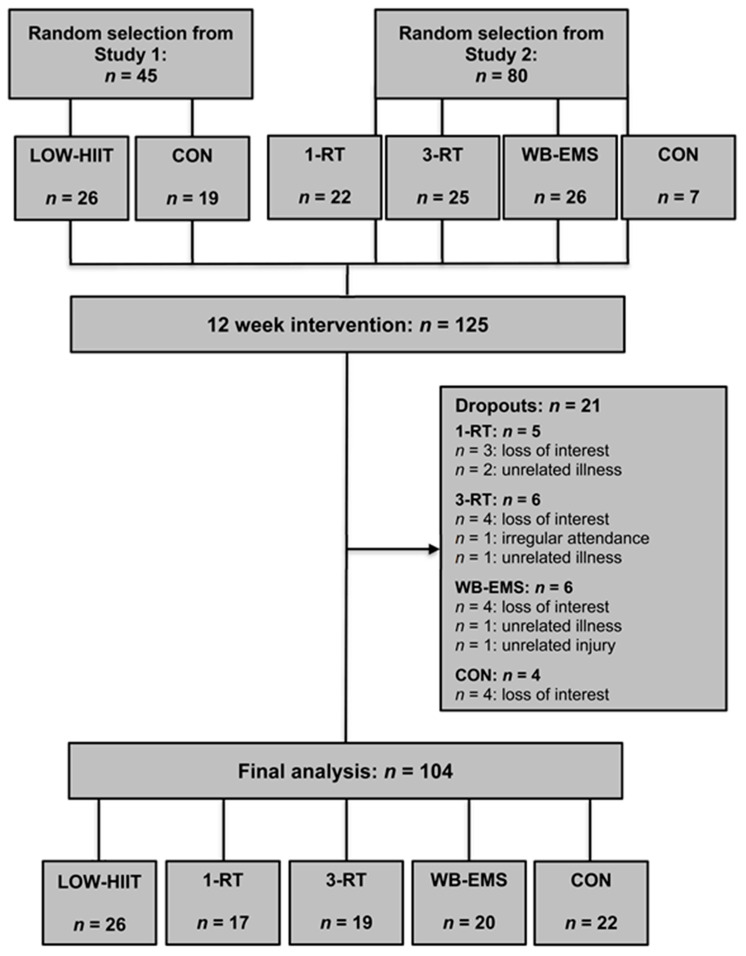
Study flow chart.

From a pool of 272 patients who participated in the two main trials (study 1: *n* = 118, study 2: *n* = 154), a total of 125 patients were included for this sub-analysis (LOW-HIIT = 26, 1-RT = 22, 3-RT = 25, WB-EMS = 26, and CON = 26). Table 1 displays the main baseline characteristics of the participants. During the intervention period, 21 patients dropped out (1-RT = 5, 3-RT = 6, WB-EMS = 6, and CON = 4). Thus, a total of 104 participants were included in the final analysis (LOW-HIIT = 26, 1-RT = 17, 3-RT = 19, WB-EMS = 20, and CON = 22). The study flow is pictured in Figure 1. There were no significant baseline differences between the five groups. Additionally, no significant effects of gender were detected in the subgroup analyses. Thus, the results of both males and females were considered together in all analyses.

### 3.2. Nutritional Analysis

Significant main time effects were detected for intakes of calories (*p* < 0.001, *ή*^2^ = 0.26), protein (*p* < 0.001, *ή*^2^ = 0.16), fat (*p* < 0.001, *ή*^2^ = 0.18), and carbohydrates (*p* < 0.001, *ή*^2^ = 0.22). Moreover, there were main time effects for protein (*p* = 0.002, *ή*^2^ = 0.11), fat (*p* < 0.001, *ή*^2^ = 0.13), and carbohydrate (*p* < 0.001, *ή*^2^ = 0.17) intakes per kg body weight. Post hoc tests showed that mean total daily calorie intake decreased in the 1-RT (−669 kcal, 95% CI: −1152 to −186 kcal, *p* = 0.010), 3-RT (−575 kcal, 95% CI: −835 to −315 kcal, *p* = 0.001), and WB-EMS group (−502 kcal, 95% CI: −840 to −164 kcal, *p* = 0.006) from baseline to follow-up. There were no significant between-group differences in calorie and macronutrient intakes. Nutritional intakes pre-intervention and during the last week of the intervention in each group are presented in Table 2.

### 3.3. Anthropometric and Body Composition Data

**Figure 2:**
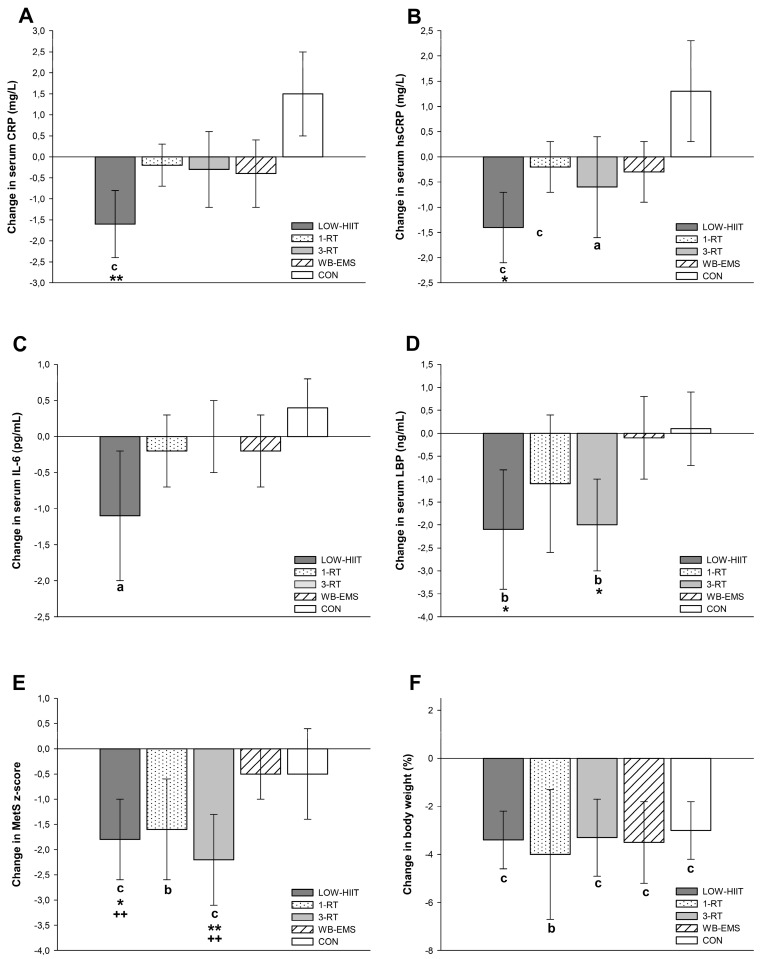
Changes in serum concentrations of C-reactive protein (**A**), high-sensitivity C-reactive protein (**B**), interleukin-6 (**C**), lipopolysaccharide-binding protein (**D**), and changes in MetS z-score (**E**) and body weight (**F**). ^a^ (*p* < 0.05), ^b^ (*p* < 0.01), ^c^ (*p* < 0.001): significant difference versus pre-intervention; * (*p* < 0.05), ** (*p* < 0.01): significant difference versus CON; ^++^ (*p* < 0.01): significant difference versus WB-EMS.

Significant main effects of time were detected for body weight (*p* < 0.001, *W* = 0.53), BMI (*p* < 0.001, *W* = 0.52), FM (*p* < 0.001, *W* = 0.53), %FM (*p* < 0.001, *W* = 0.47), skeletal muscle mass (*p* < 0.001, *W* = 0.15), body water (*p* = 0.010, *W* = 0.07), and WC (*p* < 0.001, *W* = 0.64). All groups reduced body weight (LOW-HIIT: −4.0 kg, 95% CI: −5.7 to −2.3 kg, *p* < 0.001; 1-RT: −4.8 kg, 95% CI: −7.4 to −2.1 kg, *p* = 0.002; 3-RT: −3.7 kg, 95% CI: −5.6 to −1.8 kg, *p* < 0.001; WB-EMS: −4.1 kg, 95% CI: −6.2 to −2.0 kg, *p* < 0.001; CON: −2.9 kg, 95% CI: −4.0 to −1.7 kg, *p* < 0.001), primarily due to a decrease in FM. The relative amount of weight loss did not differ significantly between groups (Figure 2). In all groups, body weight loss was accompanied by significant decreases in waist circumference and body fat mass, except for the CON group. Reductions in waist circumference were larger in the LOW-HIIT (−3.6 cm, 95% CI: −7.0 to −0.2 cm, *p* = 0.035) and 3-RT group (−4.5 cm, 95% CI: −8.3 to −0.7 cm, *p* = 0.011) in comparison to the CON group. Skeletal muscle mass decreased significantly in the WB-EMS and CON group. All group specific body composition changes are presented in Table 3.

### 3.4. Inflammatory Markers

A significant group-by-time interaction was found for CRP (*p* = 0.022, *ή*^2^ = 0.11), hsCRP (*p* = 0.028, *ή*^2^ = 0.10) and LBP (*p* = 0.011, *ή*^2^ = 0.13). Main effects of time were detected for IL-6 (*p* = 0.048, *W* = 0.37) and LBP (*p* < 0.001, *ή*^2^ = 0.14). Post-hoc tests showed significant reductions in serum concentrations of CRP (−1.6 mg/L, 95% CI: −2.4 to −0.7 mg/L, *p* < 0.001), hsCRP (−1.4 mg/L, 95% CI: −2.1 to −0.6 mg/L, *p* < 0.001), IL-6 (−1.1 pg/mL, 95% CI: −2.0 to −0.2 pg/mL, *p* = 0.020), and LBP (−2.1 ng/mL, 95% CI: −3.4 to −0.7 ng/mL, *p* = 0.004) in the LOW-HIIT group. Reductions in CRP (−3.1 mg/L, 95% CI: −5.6 to −0.5 mg/L, *p* = 0.008), hsCRP (−2.7 mg/L, 95% CI: −5.1 to −0.3 mg/L, *p* = 0.016), and LBP (−2.2 ng/mL, 95% CI: −4.3 to −0.1 ng/mL, *p* = 0.042) were greater in the LOW-HIIT group compared to the CON group (Figure 2). Post-intervention LBP serum concentrations were lower in the LOW-HIIT group compared to the 1-RT (−16.4 ng/mL, 95% CI: −22.4 to −10.5 ng/mL, *p* < 0.001), 3-RT (−15.1 ng/mL, 95% CI: −20.9 to −9.3 ng/mL, *p* < 0.001), and WB-EMS group (−11.4 ng/mL, 95% CI: −17.0 to −5.9 ng/mL, *p* < 0.001). In the 3-RT group, hsCRP (−0.6 mg/L, 95% CI: −1.2 to −0.1 mg/L, *p* = 0.044) and LBP (−2.0 ng/mL, 95% CI: −2.9 to −1.1 ng/mL, *p* < 0.001) serum concentrations were also significantly decreased post-intervention. Group-specific values of all markers of inflammation are shown in Table 4. Reductions in IL-6 serum concentration were significantly correlated with reductions in waist circumference (ρ = 0.23, *p* = 0.022). Pre- and post-intervention risk grading for the development of cardiovascular health issues related to hsCRP serum concentrations among the five groups are shown in Table 5.

### 3.5. Cardiometabolic Markers

Significant main time effects were found for SBP (*p* < 0.001, *ή*^2^ = 0.14), DBP (*p* < 0.001, *ή*^2^ = 0.12), MAB (*p* < 0.001, *ή*^2^ = 0.19), fasting glucose (*p* = 0.034, *W* = 0.04), LDL (*p* < 0.001, *ή*^2^ *=* 0.12), total cholesterol (*p* < 0.001, *ή*^2^ = 0.11) triglycerides (*p* = 0.003, *ή*^2^ = 0.08), MetS z-score (*p* < 0.001 *ή*^2^ = 0.41), and VO_2max_ (*p* < 0.001 *ή*^2^ = 0.21). Significant group-by-time interactions were detected for SBP (*p* = 0.002, *ή*^2^ = 0.16), DBP (*p* = 0.008, *ή*^2^ = 0.13), MAB (*p* < 0.001, *ή*^2^ = 0.18), MetS z-score (*p* = 0.002, *ή*^2^ = 0.16), and VO_2max_ (*p* < 0.001 *ή*^2^ = 0.22). Post-hoc tests revealed significant reductions of SBP and DBP in the LOW-HIIT (−11 mmHg, 95% CI: −16 to −6 mmHg, *p* < 0.001, and −8 mmHg, 95% CI: −11 to −4 mmHg, *p* < 0.001, respectively) and 3-RT group (−11 mmHg, 95% CI: −17 to −5 mmHg, *p* = 0.002, and −4 mmHg, 95% CI: −7 to −1 mmHg, *p* = 0.009, respectively). MAB decreased in the LOW-HIIT (−9 mmHg, 95% CI: −12 to −6 mmHg, *p* < 0.001), 1-RT (−7 mmHg, 95% CI: −14 to −1 mmHg, *p* = 0.038), and 3-RT group (−7 mmHg, 95% CI: −10 to −3 mmHg, *p* = 0.001). Reductions in MAB were larger in the LOW-HIIT (−11 mmHg, 95% CI: −19 to −3 mmHg, *p* = 0.001), 1-RT (−9 mmHg, 95% CI: −18 to −1 mmHg, *p* = 0.032), and 3-RT group (−9 mmHg, 95% CI: −18 to −1 mmHg, *p* = 0.024) in comparison to the WB-EMS group. In the 3-RT group, fasting glucose (−6 mg/dL, 95% CI: −10 to −1 mg/dL, *p* = 0.015) and triglyceride serum concentrations (−22 mg/dL, 95% CI: −49 to 5 mg/dL, *p* = 0.046) were decreased after the intervention.

Post-intervention MetS z-scores were found to be reduced in the LOW-HIIT (−1.84 units, 95% CI: −2.6 to −1.1 units, *p* < 0.001), 1-RT (−1.58 units, 95% CI: −2.6 to −0.6 units, *p* = 0.005), and 3-RT group (−2.36 units, 95% CI: −3.2 to −1.5 units, *p* < 0.001). MetS z-score reductions were larger in the LOW-HIIT and 3-RT group compared to the WB-EMS (−1.33 units, 95% CI: −2.3 to −0.4 units, *p* = 0.006, and −1.73 units, 95% CI: −2.7 to −0.7 units, *p* = 0.001, respectively) and CON group (−1.30 units, 95% CI: −2.3 to −0.2 units, *p* = 0.017, and −1.70 units, 95% CI: −2.8 to −0.6 units, *p* = 0.005, respectively) (Figure 2). VO_2max_ only significantly increased in the LOW-HIIT group (3.0 mL/kg/min, 95% CI: 2.0 to 4.0 mL/kg/min, *p* < 0.001). The increase in VO_2max_ was significantly larger in the LOW-HIIT group compared to the WB-EMS (2.2 mL/kg/min, 95% CI: 0.2 to 4.1 mL/kg/min, *p* = 0.019) and CON group (3.4 mL/kg/min, 95% CI: 1.5 to 5.2 mL/kg/min, *p* < 0.001) (Figure 2). Group-specific values of all cardiometabolic indices are shown in Table 6. MetS z-score reductions were significantly associated with waist circumference decreases (ρ = 0.45, *p* < 0.001). Moreover, there was a significant correlation between MetS z-score reductions and reductions in serum concentrations of CRP (*r* = 0.24, *p* = 0.014) and hsCRP (*r* = 0.25, *p* = 0.012). Changes in VO_2max_ were significantly correlated to changes in CRP (*r* = 0.32, *p* = 0.001) and hsCRP (*r* = 0.32, *p* = 0.001).

## 4. Discussion

Although there is sound evidence that regular cardiovascular [19,20] and resistance [20,21] training can have beneficial impact on chronic inflammation, there is still a lack of data regarding the effects of (very) low-volume exercise modalities, which have gained increasing popularity in recent years due to their greater time-efficiency compared to traditional exercise methods. To our knowledge, this study was the first to comprehensively investigate and compare the chronic responses of inflammatory markers to LOW-HIIT, 1-RT, and WB-EMS in a cohort of obese MetS patients. As presented in Figure 2, the key findings were as follows: (i) all exercise groups and the CON group experienced a similar relative weight loss during the period of caloric restriction by an average of 3.6%, (ii) serum concentrations of CRP, hsCRP, IL-6, and LBP only significantly decreased in the LOW-HIIT group, (iii) among the RT modalities, only traditional 3-RT had an impact on inflammation as it became evident by significant reductions in serum concentrations of hsCRP and LBP, and (iv) MetS severity score only improved in the LOW-HIIT, 1-RT, and 3-RT groups, while WB-EMS and CON did not have a significant effect on cardiometabolic risk profile.

The average weight loss achieved in all five study groups during the 12-week intervention period (–3.6% of baseline body weight) is in line with the average relative weight loss values (~3%) reported in most obesity treatment programs targeting lifestyle changes [61]. It is well established that weight loss is a critical factor to improve chronic inflammation [62] and cardiometabolic health [13,14]. In this context, a weight loss of at least 5% is frequently advocated in the literature as a threshold that needs to be reached to induce clinically meaningful improvements in health outcomes [63]. Thus, the lack of significant effects on inflammatory markers in the inactive CON group may indicate that a reduction of body weight of less than ~5% is not sufficient to induce a meaningful reduction in chronic inflammation if weight loss is achieved only through caloric restriction. However, it has also been pointed out in previous research that obesity treatments should not exclusively focus on weight loss alone, but rather target on changes in body composition (in particular, a reduction in visceral fat mass) [64,65]. Although not statistically significant, we found that post-intervention CRP concentrations even tended to increase in the CON group, which may be potentially associated with less beneficial changes in body composition compared with the exercise groups. In addition to the need to reduce fat mass, which is considered a major source of obesity-related inflammation [8,9,10,11,12], it is also widely recognized that preservation (or ideally increasing) of skeletal muscle mass during weight loss should be a further goal in obesity treatment. Besides their key function to produce body movements, skeletal muscles also represent the body’s largest metabolically active tissue with substantial influence on glucose and insulin regulation, and basal metabolic rate [66,67]. Additionally, a current meta-analysis has demonstrated that skeletal muscle mass is significantly inversely related with inflammatory markers [68]. Moreover, it has been suggested that exercise-induced cytokines that are released from the muscles during contractions have direct anti-inflammatory effects and serve as a mechanism to improve cardiometabolic health [69]. Although caloric restriction is a key measure to reduce weight, it has also been well documented that weight loss achieved through caloric deficit alone is usually accompanied by a loss in skeletal muscle mass by amounts ranging between ~2 and 10% [70]. In this context, it is important to note that skeletal muscle mass declined to a smaller (although not statistically significant) degree in the LOW-HIIT (−1.5%) and 3-RT (−0.3%) groups than in the CON group (−2.3%), while reductions in waist circumference (as an indicator of visceral fat mass) were significantly greater (LOW-HIIT: −3.6 cm and 3-RT: −4.5 cm), compared to the CON group.

Based on the fact that there were no significant between-group differences in weight loss (in particular when the exercise groups are compared versus the inactive CON group), it may be speculated that two weekly sessions of low-volume exercise (LOW-HIIT, 1-RT, or WB-EMS) as well as two sessions of 3-RT per week have only made a small contribution to the overall negative energy balance. Accordingly, it has been reported that energy expenditures during low-volume exercise modalities, including LOW-HIIT [71], 1-RT [72] and WB-EMS [73], are quite small, ranging around 120–150 kcal per exercise session. However, it has also been documented that HIIT exercises are particularly effective to stimulate excess post-exercise oxygen consumption (EPOC), a valid measure to quantify elevated energy expenditure after cessation of exercise, which has been associated with higher fat loss over a longer period of time [74,75,76]. Furthermore, it has recently been shown that both HIIT and traditional RT resulted in increased resting metabolic rate for at least 14 h post-exercise in healthy women [77]. Additionally, it has been pointed out that simultaneous but opposing adaptations in fat tissue and muscle mass may appear in response to exercise that cannot be properly detected when only body weight and BMI are considered but by changes in body composition and waist circumference, respectively [65]. Recently, it has also been reported that there is emerging evidence for the potential of HIIT (although primarily considered as cardiovascular training) in promoting skeletal muscle anabolism [78]. Moreover, it is important to note that exercise per se evokes a wide range of beneficial physiological changes linked to improved health outcomes that go well beyond simple weight loss [79,80].

Several meta-analyses have already revealed the potential of HIIT to reduce markers of inflammation in various populations, including healthy individuals [81,82], overweight and obese cohorts [44], and patients with cardiometabolic disorders [81,82,83,84]. However, currently, data on the effects of low-volume HIIT (i.e., protocols with a maximal session duration of ≤30 min and ≤10 min of intense exercise, respectively, as previously defined [28]) are still very sparse. Kelly et al. [85] investigated the impact of a low-volume HIIT protocol (10 × 1 min intervals at 90% HR_max_, total session time: 24 min) that was performed for 2 weeks on various physiological parameters, including body composition, glucose control, and inflammatory markers in overweight and obese men. The authors failed to find significant changes in any of the outcome measures, which may be due to the short intervention duration and the small total number of exercise sessions (study group 1: 6 sessions, and study group 2: 4 sessions within 2 weeks, respectively). Comparable to our results (−30.6%), Asle Mohammadi Zadeh et al. [86] observed that 24 weeks of low-volume HIIT (10 × 1 min intervals at 75–90% HR_max_) combined with a low-carbohydrate diet resulted in a significant reduction in IL-6 concentration (−32.1%). In comparison to the low-volume HIIT protocol employed in the previous investigations [85,86], it is important to highlight that the time effort for our very low-volume HIIT protocol (28 min/week) was ~50% lower. Given that “lack of time” is a major obstacle to regular exercise for obese individuals [26], it is a key result of our investigation that engaging in less than 30 min of exercise per week is still effective to induce significant improvements in inflammation status and cardiometabolic indices in obese patients with MetS, which may be an important factor to enable greater adherence to exercise regimes over time. Although data suggest that higher-intensity exercise may also be more effective in reducing CRP levels compared to lower-intensity exercises [87], recent meta-analyses did not provide clear evidence whether HIIT is generally superior to traditional MICT regarding the effects on inflammation status [82,84]. We speculate therefore, that both modalities may provide similar benefits in this respect, with HIIT, however, requiring less time compared to MICT. In accordance with the current literature [88], we recommend that ideally, both HIIT and MICT should be incorporated into a well-rounded exercise treatment program for obese individuals to achieve optimal results as both exercise modalities are associated with specific physiological adaptations, provided that patients are willing to invest sufficient time in exercise.

The significant reductions in some major inflammatory markers in the LOW-HIIT group can likely provide benefits of clinical relevance since CRP [89], IL-6 [90], and LBP [91,92] concentrations are closely linked to MetS and associated secondary diseases and health risks. In line with the literature, we observed a significant correlation between changes in CRP and hsCRP, respectively, and MetS severity (MetS z-score) in our patients. Remarkably, the observed reduction in serum concentration of hsCRP (−35%) following LOW-HIIT was comparable to effects achieved in pharmacological interventions [60,93]. In 2017, for example, the CANTOS study received great attention, showing that the IL-1β inhibitor canakinumab administered subcutaneously in doses of 150 mg every 3 months for 48 months lowered hs-CRP levels by 35% [94]. Additionally, it is noteworthy that in the LOW-HIIT group, the number of patients with hsCRP serum concentrations of >3 mg/L (i.e., a level that is associated with high risk for developing cardiovascular problems [60]) was significantly reduced by ~50%. Likewise, a considerable number of patients had reduced their hsCRP concentrations to a level of <1 mg/L (i.e., low-risk for developing cardiovascular problems [60]) (Table 6), further pointing to the clinical relevance of the anti-inflammatory effects of LOW-HIIT. In this context, we note that approximately a quarter of patients in the LOW-HIIT group still displayed hsCRP levels of >3 mg/L post-intervention. We speculate, therefore, that such non-responders and patients with substantially elevated baseline CRP levels, respectively, may require further support to reduce chronic inflammation. Intensified measures to reduce chronic inflammation may include higher volumes of exercise; higher frequencies of exercise sessions and/or combined aerobic and resistance training programs [44,95]; more targeted nutritional modifications, e.g., a Mediterranean-based diet, which has shown particular anti-inflammatory effects [96]; or if lifestyle changes alone do not provide sufficient results, additional pharmacological treatment.

In accordance with the literature [44,97], LOW-HIIT (a type of cardiovascular exercise) showed superior efficacy in reducing inflammatory markers compared with the RT groups. It has been reported that cardiovascular-based exercise modalities, such as MICT or HIIT, primarily promote a reduction in pro-inflammatory markers such as CRP and IL-6, while RT rather triggers an increase in anti-inflammatory cytokines (e.g., IL-10) [44]. A large cohort study has shown, for example, that the degree of cardiorespiratory fitness (i.e., VO_2max_) was inversely associated with chronic inflammation and that this relationship was strongly mediated but not entirely explained by changes in body weight and body composition [98]. In line with this, we found that the improvements in VO_2max_, which only occurred in the LOW-HIIT group, were significantly inversely correlated with reductions in CRP and hsCRP, respectively. The finding that among the three RT groups, only traditional 3-RT had a positive effect on inflammatory markers may indicate that RT-based exercise modalities apparently require a higher volume to produce significant improvements in chronic inflammation.

The beneficial effects of our LOW-HIIT and RT protocols on cardiometabolic risk indices have already been reported in previous research from our group [34,35,39] and were now confirmed in this sub-collective of obese MetS patients in the present investigation. The previous results are extended by the finding that the observed improvements in MetS severity and VO_2max_ were significantly related to reductions in inflammatory markers. Accordingly, LOW-HIIT had the strongest effects on cardiometabolic health indices, when considering both changes in VO_2max_ and the MetS z-score together. The improvements in MetS z-score were mainly due to marked reductions in blood pressure and a decrease in waist circumference. In accordance with previous research, showing that cardiovascular-based exercise is more effective in improving blood pressure compared to RT [99], we found that the LOW-HIIT group experienced the largest reduction in MAB. However, 1-RT and 3-RT also provided significant anti-hypertensive benefits that are comparable with pharmacological treatment effects [100], previously associated with a lowered risk of CVD and mortality [101]. Compared to other low-volume HIIT studies in various populations, as recently summarized by Sabag et al. [71], we did not observe significant changes in cardiometabolic blood indices in the LOW-HIIT group, which may potentially be attributed to the extremely low exercise volume of our protocol. However, previously we have found significant reductions in LDL serum concentrations after an 8-week intervention of LOW-HIIT in untrained normal-weight individuals [58]. Thus, we assume that our LOW-HIIT protocol can evoke positive alterations in blood lipids in metabolically less compromised individuals, while obese MetS patients, who typically display unfavorable metabolism abnormalities (e.g., altered substrate utilization [102]), may require higher volumes of exercise to achieve significant improvements in cardiometabolic blood markers. In accordance with the literature, showing that increased volume of RT via an increased number of sets has a larger impact on blood lipids [103], we observed that among the RT modalities, only 3-RT significantly lowered serum triglyceride concentrations. It is important to note, however, that patients in the 1-RT also experienced a significant reduction in MetS z-score. This finding is important, since the MetS z-score has been found to be more sensitive to predict the patient’s future CVD risk compared to simply summing up single cardiometabolic risk components [104]. Consequently, as previously reported [39], the present data indicate that 1-RT can be considered an effective and time-saving exercise modality to improve cardiometabolic risk outcomes in obese MetS patients.

Although widely advertised as a viable, more time-efficient alternative to traditional RT in commercial fitness facilities, the scientific basis for the efficacy of WB-EMS in improving cardiometabolic health outcomes is still relatively sparse and inconclusive. Previous research, including work from our laboratory, has documented either significant improvements in the MetS z-score [43,105] or lack of effects [39,106]. The findings in the sub-group of patients in the present investigation are in line with our data previously obtained from a larger sample [39], indicating that traditional RT (even when matched in terms of time-effort, i.e., WB-EMS vs. 1-RT) appears to be superior to WB-EMS in improving cardiometabolic health markers in individuals with cardiometabolic alterations. Likewise, WB-EMS failed to improve inflammatory markers. As previously speculated, the differences in the efficacy between WB-EMS and traditional RT may potentially be due to a greater stimulation of skeletal muscle protein synthesis by RT and/or different effects on muscle fiber adaptations [39]. Nevertheless, we note that WB-EMS had positive effects on body composition, including waist circumference, which is regarded as a crucial single risk component of MetS [98]. According to previous research [107], a reduction of 3 cm in waist circumference, as observed in WB-EMS group, can be regarded clinically relevant. Thus, although less effective compared to traditional RT, WB-EMS can be considered as an alternate muscular exercise modality if specific contraindications to RT are evident (e.g., orthopaedic conditions that preclude heavy weightlifting).

Finally, we note some limitations of our study that should be considered. First, the evaluation of patients’ dietary changes during the intervention period was based on self-reported food records. Although patients were strongly motivated to reduce their body weight and to contribute to improving their health status, self-reported measures may be linked to some bias, including social desirability or lack of memory. We suspect, however, that the thorough instruction and close supervision during the recording phases should have substantially decreased the magnitude of potential bias. Moreover, we found that the objectively measured body weight reduction matched quite well with patients’ self-reported reduction in caloric intake. Second, while we used the 3-RT group as an “active” high-volume control group to compare against the low-volume exercise modalities, it could be critically noted that we did not additionally include a high-volume cardiovascular-based exercise group (i.e., MICT). In this context, we point out that the effects of HIIT versus MICT on cardiometabolic [32,33] and inflammatory markers [82,84] have already been comprehensively compared in previous research, including a study from our group [58], and that the present investigation primarily aimed to compare the efficacy of low-volume exercise modalities. We acknowledge, however, that the inclusion of a further MICT group would have potentially provided further information in evaluating the role of low-volume exercise protocols in obesity treatment compared to more traditional exercise modalities. Third, as previously mentioned, the interpretation of our results is limited by the fact that we did not assess specific anti-inflammatory blood markers which future research may wish to address. Finally, we emphasize that the exercise interventions were performed in a well-controlled laboratory setting over a period of 12 weeks with close supervision during all training sessions. Further research will be needed to determine the long-term effects of low-volume exercise modalities in real-world conditions.

## 5. Conclusions

To our knowledge, this was the first study to compare the effects of the popular exercise methods LOW-HIIT, 1-RT, and WB-EMS on markers of inflammation and cardiometabolic health in a cohort of obese MetS patients. Our results demonstrate that LOW-HIIT effectively improved several markers of inflammation and cardiometabolic health in this high-risk collective, and thus can be regarded as a viable tool in the treatment of low-grade chronic inflammation and MetS. Overall, LOW-HIIT appears to provide more favorable effects on chronic inflammation and cardiometabolic health outcomes compared to low-volume RT modalities, while WB-EMS was found to be the least effective. Apparently, exercise programs based on RT require a higher training volume (i.e., multiple-sets) to induce beneficial changes in inflammation status, while single-set RT can be effective to produce improvements in cardiometabolic health status. The practical take-home message from this study is that health professionals working with obese MetS patients can be encouraged to incorporate low-volume exercise modalities into treatment programs. Among the different low-volume exercise modalities, cardiovascular-based exercises, such as LOW-HIIT, should be emphasized over low-volume RT-based modalities due to the superior effects on inflammation. However, RT can also play an important role in improving cardiometabolic health and ideally, both cardiovascular- and RT-based modalities should be combined, along with an adequate dietary modification, to achieve optimal treatment results.

## Figures and Tables

**Table 1:** Baseline characteristics of all included patients.

Variable	LOW-HIIT (*n* = 26)	1-RT (*n* = 22)	3-RT (*n* = 25)	WB-EMS (*n* = 26)	CON (*n* = 26)
Gender (f/m)	10/16	16/6	15/10	18/8	18/8
Age (years)	50.6 ± 11.3	55.2 ± 12.1	52.7 ± 11.5	52.7 ± 12.5	49.0 ± 15.1
BMI (kg/m^2^)	37.8 ± 6.6	36.8 ± 7.4	40.1 ± 9.0	37.2 ± 4.0	38.0 ± 6.3
CRP (mg/L)	5.0 ± 3.4	6.1 ± 5.8	5.7 ± 6.3	4.1 ± 2.8	4.2 ± 3.3
MetS z-score	2.9 ± 3.9	3.4 ± 4.8	3.3 ± 4.3	2.6 ± 2.3	2.2 ± 3.1

Values are shown as mean ± SD. f = females, m = males, BMI = body mass index, CRP = C-reactive protein, MetS z-score = metabolic. syndrome z-score.

**Table 2:** Daily nutritional intakes before the intervention and during the last week of intervention.

Variable	LOW-HIIT (*n* = 26)		1-RT (*n* = 17)		3-RT (*n* = 19)		WB-EMS (*n* = 20)		CON (*n* = 22)	
	Pre	Post	Pre	Post	Pre	Post	Pre	Post	Pre	Post
Energy (kcal/day)	2228 ± 896	1974 ± 801	2342 ± 907	1673 ± 520 ^a^	2344 ± 622	1769 ± 514 ^b^	2418 ± 695	1916 ± 415 ^b^	2237 ± 856	1798 ± 783
Protein (g/day)	92 ± 46	90 ± 31	102 ± 46	78 ± 28 ^a^	108 ± 41	80 ± 27 ^b^	96 ± 30	89 ± 31	94 ± 30	82 ± 33
Protein (g/kg/day)	0.8 ± 0.3	0.8 ± 0.3	0.9 ± 0.3	0.7 ± 0.3	1.0 ±0.4	0.8 ± 0.3	1.0 ± 0.5	0.8 ± 0.3	1.0 ± 0.3	0.9 ± 0.3
Fat (g/day)	89 ± 41	77 ± 36	97 ± 40	71 ± 28	96 ± 31	72 ± 26 ^b^	99 ± 40	80 ± 19	99 ± 57	70 ± 43
Fat (g/kg/day)	0.8 ± 0.3	0.7 ± 0.3	1.0 ± 0.4	0.8 ± 0.3	1.0 ± 0.4	0.8 ± 0.4	0.9 ± 0.4	0.8 ± 0.3	1.0 ± 0.5	0.7 ± 0.5
CHO (g/day)	216 ± 97	206 ± 92	237 ± 92	160 ± 51 ^b^	216 ± 58	170 ± 52 ^b^	249 ± 82	184 ± 61 ^a^	210 ± 76	180 ± 84
CHO (g/kg/day)	1.9 ± 0.7	1.8 ± 0.8	2.4 ± 0.7	1.8 ± 0.7 ^a^	2.4 ± 0.8	1.7 ± 0.7 ^b^	2.0 ± 0.7	1.6 ± 0.6 ^a^	2.1 ± 0.9	1.9 ± 1.0
Fibres (g/day)	23 ± 9	23 ± 11	24 ± 11	21 ± 8	23 ± 7	19 ± 7	22 ± 10	18 ± 7	24 ± 13	23 ± 10

Values are shown as mean ± SD. CHO = carbohydrates. ^a^ (*p* < 0.05), ^b^ (*p* < 0.01): significant difference versus pre-intervention.

**Table 3:** Anthropometric and body composition data before and after the intervention.

Variable	LOW-HIIT (*n* = 26)		1-RT (*n* = 17)		3-RT (*n* = 19)		WB-EMS (*n* = 20)		CON (*n* = 22)	
	Pre	Post	Pre	Post	Pre	Post	Pre	Post	Pre	Post
Weight (kg)	117.0 ± 26.1	113.0 ± 25.2 ^c^	102.9 ± 27.7	98.1 ± 26.0 ^b^	114.0 ± 30.1	110.3 ± 30.1 ^c^	106.1 ± 17.9	101.9 ± 14.6 ^c^	104.4 ± 20.5	101.5 ± 21.5 ^c^
BMI (kg/m^2^)	37.8 ± 6.6	36.5 ± 6.4 ^c^	36.5 ± 7.4	34.8 ± 7.1 ^b^	38.8 ± 8.1	37.5 ± 7.5 ^b^	37.3 ± 4.1	35.9 ± 3.2 ^c^	36.6 ± 5.2	35.5 ± 5.5 ^c^
FM (kg)	49.8 ± 14.8	46.3 ± 14.3 ^c,^*	47.7 ± 16.1	43.9 ± 14.8 ^b^	51.8 ± 16.6	48.0 ± 16.1 ^c,^*	46.0 ± 8.2	43.1 ± 6.1 ^b^	47.8 ± 11.5	45.7 ± 12.7
FM (%)	42.3 ± 7.6	40.7 ± 8.1 ^c,^*	45.8 ± 5.4	44.2 ± 5.7 ^b^	45.3 ± 6.6	43.3 ± 7.3 ^c,^*	43.6 ± 5.1	42.6 ± 5.0 ^b^	46.1 ± 7.0	44.9 ± 7.5
SMM (kg)	33.3 ± 9.0	32.8 ± 8.9	26.5 ± 7.8	25.8 ± 7.8	30.7 ± 10.4	30.6 ± 10.6	29.2 ± 7.0	28.3 ± 6.3 ^b^	27.5 ± 7.9	26.9 ± 7.9 ^c^
TBW (L)	50.0 ± 11.7	49.5 ± 11.7	41.6 ± 9.9	40.8 ± 9.9	46.7 ± 12.4	46.7 ± 12.8 ^c^	44.8 ± 9.1	43.9 ± 9.1	44.8 ± 10.8	43.9 ± 10.5
Waist (cm)	116 ± 19	110 ± 18 ^c,^*	111 ± 16	106 ± 14 ^c^	116 ± 18	111 ± 17 ^c,^*	114 ± 10	111 ± 9 ^b^	109 ± 11	107 ± 11

Values are shown as mean ± SD. BMI = body mass index, FM = fat mass, SMM = skeletal muscle mass, TBW = total body water. ^b^ (*p* < 0.01), ^c^ (*p* < 0.001): significant difference versus pre-intervention; * (*p* < 0.01): significant difference versus CON.

**Table 4:** Inflammatory markers before and after the intervention.

Variable	LOW-HIIT (*n* = 26)		1-RT (*n* = 17)		3-RT (*n* = 19)		WB-EMS (*n* = 20)		CON (*n* = 22)	
	Pre	Post	Pre	Post	Pre	Post	Pre	Post	Pre	Post
CRP (mg/L) ^#^	5.0 ± 3.4	3.4 ± 2.7 ^b,^*	4.7 ± 4.8	4.5 ± 4.7	4.0 ± 3.3	3.7 ± 2.9	3.8 ± 2.4	3.4 ± 2.4 ^b^	3.9 ± 2.7	5.4 ± 8.0
hsCRP (mg/L) ^#^	4.1 ± 3.1	2.7 ± 2.3 ^b,^*	4.0 ± 4.6	3.8 ± 4.6	3.3 ± 3.2	2.7 ± 2.5 ^a^	2.9 ± 2.0	2.6 ± 2.1	3.1 ± 2.4	4.4 ± 7.3
IL-1β (pg/mL)	6.3 ± 4.3	6.4 ± 3.8	9.7 ± 8.4	11.8 ± 9.8	9.8 ± 5.6	10.7 ± 6.5	10.0 ± 5.6	11.1 ± 8.3	6.7 ± 4.1	6.2 ± 3.8
IL-6 (pg/mL)	3.6 ± 3.0	2.5 ± 1.6 ^a^	2.8 ± 1.6	2.6 ± 1.7	2.6 ± 1.1	2.6 ± 1.6	2.5 ± 1.3	2.3 ± 1.2	3.1 ± 1.6	3.4 ± 3.0
IFNγ (pg/mL)	9.5 ± 6.8	8.8 ± 4.2	7.0 ± 4.1	6.4 ± 4.4	10.3 ± 9.4	9.1 ± 9.0	7.4 ± 2.9	7.2 ± 3.2	8.1 ± 4.5	7.5 ± 4.3
Adiponectin [µ/mL)	2.3 ± 1.3	2.2 ± 1.2	3.8 ± 2.7	3.9 ± 3.1	3.6 ± 3.0	3.2 ± 2.3	2.5 ± 1.5	2.4 ± 1.2	2.6 ± 1.3	2.6 ± 1.3
LBP (ng/mL) ^#^	13.2 ± 2.5	11.1 ± 3.1 ^b,^*^,§^	29.1 ± 7.6	28.0 ± 7.1	28.2 ± 3.6	26.3 ± 5.6 ^c^	23.6 ± 5.8	23.5 ± 5.8	16.9 ± 8.9	17.0 ± 9.4

Values are shown as mean ± SD. CRP = C-reactive protein, hsCRP = high-sensitivity C-reactive protein, IL-1 = interleukine-1, hsIL-6 = high-sensitivity inter-leukine-6, INFα = interferone-alpha, LBP = lipopolysaccharide-binding protein. ^a^ (*p* < 0.05), ^b^ (*p* < 0.01), ^c^ (*p* < 0.001): significant difference versus pre-intervention; * (*p* < 0.05): significant difference vs. CON; ^§^ (*p* < 0.001): significant difference versus 1-RT, 3-RT, WB-EMS; ^#^ (*p* < 0.05): significant group-by-time interaction.

**Table 5:** Cardiovascular disease risk distribution related to hsCRP concentrations before and after the intervention.

Variable	LOW-HIIT (*n* = 26)		1-RT (*n* = 17)		3-RT (*n* = 19)		WB-EMS (*n* = 20)		CON (*n* = 22)	
	Pre	Post	Pre	Post	Pre	Post	Pre	Post	Pre	Post
Low risk (<1 mg/L)	1 (3.8)	6 (23.1)	4 (23.5)	6 (35.3)	3 (15.8)	4 (21.1)	3 (15.0)	3 (15.0)	1 (4.5)	3 (13.6)
Intermediate risk	10 (38.5)	13 (50.0)	6 (25.3)	3 (17.6)	6 (31.6)	7 (36.8)	9 (45.0)	10 (50.0)	13 (59.1)	8 (36.4)
(1–3 mg/L)										
High risk	15 (57.7)	7 (26.9)	7 (41.2)	8 (47.1)	7 (52.6)	8 (42.1)	8 (40.0)	7 (35.0)	8 (36.4)	11 (50.0)
(>3 mg/L)										

Values are shown as total numbers and percentages in parentheses. Cardiovascular risk grading according to Cardoso and Paulos [60].

**Table 6:** Cardiometabolic markers before and after the intervention.

Variable	LOW-HIIT (*n* = 26)		1-RT (*n* = 17)		3-RT (*n* = 19)		WB-EMS (*n* = 20)		CON (*n* = 22)	
	Pre	Post	Pre	Post	Pre	Post	Pre	Post	Pre	Post
VO_2max_ (mL/kg/min) ^#^	22.6 ± 5.5	25.6 ± 5.6 ^c,+^	21.0 ± 5.4	22.2 ± 4.6	21.0 ± 5.1	22.0 ± 4.3	21.0 ± 5.7	21.7 ± 5.4	20.6 ± 7.4	20.2 ± 7.9
MetS z-score ^#^	2.9 ± 3.8	1.1 ± 3.0 ^c,+^	2.1 ± 3.8	0.5 ± 3.0 ^b^	2.8 ± 4.1	0.5 ± 3.9 ^c,+^	2.3 ± 2.3	1.8 ± 2.3	2.0 ± 3.1	1.3 ± 3.0
SBP (mmHg) ^#^	144 ± 17	133 ± 11 ^c^	148 ± 17	138 ± 12	142 ± 17	132 ± 16 ^b^	134 ± 14	137 ± 11	138 ± 13	137 ± 11
DBP (mmHg) ^#^	94 ± 11	86 ± 8 ^c^	92 ± 16	87 ± 10	87 ± 9	83 ± 9 ^b^	86 ± 9	88 ± 10	89 ± 9	87 ± 7
MAB (mmHg) ^#^	111 ± 11	102 ± 7 ^c^	111 ± 14	104 ± 9 ^a^	106 ± 11	99 ± 11 ^b^	102 ± 9	104 ± 9	105 ± 9	104 ± 7
Glucose (mg/dL)	101 ± 18	100 ± 12	96 ± 16	96 ± 12	105 ± 13	99 ± 14 ^a^	104 ± 12	102 ± 14	98 ± 15	95 ± 16
Triglycerides (mg/dL)	132 ± 56	128 ± 44	126 ± 43	119 ± 28	146 ± 89	124 ± 56 ^a^	133 ± 59	118 ± 36	148 ± 73	130 ± 64
Cholesterol (mg/dL)	214 ± 33	213 ± 35	228 ± 29	218 ± 33	227 ± 55	216 ± 45 ^b^	217 ± 35	210 ± 31	235 ± 42	222 ± 33 ^a^
HDL (mg/dL)	49 ± 10	48 ± 11	59 ± 19	59 ± 18	59 ± 17	58 ± 15	53 ± 14	52 ± 13	56 ± 12	54 ± 12
LDL (mg/dL)	144 ± 27	143 ± 27	148 ± 23	138 ± 22 ^a^	144 ± 39	137 ± 34	143 ± 28	137 ± 24	154 ± 33	147 ± 26

Values are shown as mean ± SD. SBP = systolic blood pressure, DBP = diastolic blood pressure, MAB = mean arterial blood pressure, HDL = high-density lipoprotein cholesterol, LDL = low-density lipoprotein cholesterol, LBP = lipopolysaccharide-binding protein. ^a^ (*p* < 0.05), ^b^ (*p* < 0.01), ^c^ (*p* < 0.001): significant difference versus pre-intervention; ^+^ (*p* < 0.05): significant difference versus WB-EMS and CON; ^#^ (*p* < 0.01): significant group-by-time interaction.

## Data Availability

The datasets generated and analyzed during the current study are not publicly available but are available from the corresponding author on reasonable request.
